# Continuing Professional Development – Medical Imaging

**DOI:** 10.1002/jmrs.696

**Published:** 2023-06-23

Maximise your CPD by reading the following selected article and answer the five questions. Please remember to self‐claim your CPD and retain your supporting evidence. Answers will be available via the QR code and online at www.asmirt.org/news-and-publications/jmrs, as well as published in JMRS – Volume 70, Issue 4 December 2023.

## Medical Imaging – Original Article

### Entrustable professional activities of graduate accredited General Medical Sonographers in Australia – Industry perceptions

Edwards C, Perry R, Chester D, Childs J. (2023). *J Med Radiat Sci*. https://doi.org/10.1002/jmrs.676

1. Which of the following best describes an entrustable professional activity (EPA) based on the authors' explanation?
  - A small unit of competency sitting within a specific examination.
  - A group of specific examinations that form a larger unit of competencies.
  - A group of non‐technical competencies which are required to meet a larger critical practice unit.
  - An examination type which sits within a larger critical practice unit and is defined by the work activity.
2. The stakeholder survey results demonstrated what percentage agreement for the existing Australian Sonographers Accreditation Registry (ASAR) EPAs?
  - >85%
  - >80%
  - >75%
  - >70%
3. The survey data shows that stakeholder opinion supports the addition of which critical practice unit?
  - Paediatrics
  - Paediatric abdomen (renal)
  - Paediatric abdomen (appendix)
  - Pyloric stenosis
4. Which ASAR critical practice unit contains activities that overlap with the Superficial Parts unit?
  - Obstetrics & Gynaecology
  - Musculoskeletal
  - Breast
  - Abdomen
5. Which of the following statements about competency assessment is supported by the authors?
  - Assessing clinical competencies by smaller iterative units of behaviours is educational best practice.
  - Overall ratings in assessment tasks are a better reflection of competence than detailed marking criteria.
  - University based assessments should primarily focus on quantitative task competence.
  - Work readiness is best evaluated by education providers.

Recommended further reading:1

Carmichael
MA
, 
Bridge
P
. Clinical perceptions of radiation therapy undergraduate competency standards. J Med Radiat Sci.
2014; Dec;61(4): 241–245. doi: 10.1002/jmrs.82
2559897710.1002/jmrs.82PMC42821052

Hodges
B
. Assessment in the post‐psychometric era: learning to love the subjective and collective. Med Teach.
2013; Jul;35(7): 564–568. doi: 10.3109/0142159X.2013.789134
2363140810.3109/0142159X.2013.7891343

Cate
OT
. A primer on entrustable professional activities. Korean J Med Educ.
2018; Mar;30(1): 1–10. doi: 10.3946/kjme.2018.76
2951060310.3946/kjme.2018.76PMC5840559

## Answers

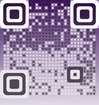

Scan this QR code to find the answers, or visit www.asmirt.org/news-and-publications/jmrs.
